# Novel putative causal mutations associated with fat traits in Nellore cattle uncovered by eQTLs located in open chromatin regions

**DOI:** 10.1038/s41598-024-60703-5

**Published:** 2024-05-02

**Authors:** Ingrid Soares Garcia, Bárbara Silva-Vignato, Aline Silva Mello Cesar, Juliana Petrini, Vinicius Henrique da Silva, Natália Silva Morosini, Carolina Purcell Goes, Juliana Afonso, Thaís Ribeiro da Silva, Beatriz Delcarme Lima, Luan Gaspar Clemente, Luciana Correia de Almeida Regitano, Gerson Barreto Mourão, Luiz Lehmann Coutinho

**Affiliations:** 1 Department of Animal Science, College of Agriculture “Luiz de Queiroz”, University of São Paulo, Piracicaba, SP Brazil; 2 Department of Agroindustry, Food and Nutrition, College of Agriculture “Luiz de Queiroz”, University of São Paulo, Piracicaba, SP Brazil; 3 Embrapa Pecuária Sudeste, São Carlos, SP Brazil

**Keywords:** Biotechnology, Animal biotechnology, Genomics

## Abstract

Intramuscular fat (IMF) and backfat thickness (BFT) are critical economic traits impacting meat quality. However, the genetic variants controlling these traits need to be better understood. To advance knowledge in this area, we integrated RNA-seq and single nucleotide polymorphisms (SNPs) identified in genomic and transcriptomic data to generate a linkage disequilibrium filtered panel of 553,581 variants. Expression quantitative trait loci (eQTL) analysis revealed 36,916 cis-eQTLs and 14,408 trans-eQTLs. Association analysis resulted in three eQTLs associated with BFT and 24 with IMF. Functional enrichment analysis of genes regulated by these 27 eQTLs revealed noteworthy pathways that can play a fundamental role in lipid metabolism and fat deposition, such as immune response, cytoskeleton remodeling, iron transport, and phospholipid metabolism. We next used ATAC-Seq assay to identify and overlap eQTL and open chromatin regions. Six eQTLs were in regulatory regions, four in predicted insulators and possible CCCTC-binding factor DNA binding sites, one in an active enhancer region, and the last in a low signal region. Our results provided novel insights into the transcriptional regulation of IMF and BFT, unraveling putative regulatory variants.

## Introduction

Backfat thickness (BFT) and intramuscular fat (IMF) are significant economic traits for the beef industry, impacting carcass and meat quality. Associated with beef sensorial quality, IMF is positively correlated with meat tenderness and juiciness, affecting its flavor, thus resulting in consumer satisfaction and repurchase decision^1^. BFT significantly influences carcass quality and yield, as an appropriate subcutaneous fat cover is essential for mitigating issues such as cold shortening and evaporative weight loss during cooling^2^. There is also a positive correlation between BFT and IMF^3^, making them interesting traits to study simultaneously.

Large-scale RNA-seq and high-throughput genotyping technologies led to genome-wide identification of regulatory variants associated with gene expression, also known as expression quantitative trait loci (eQTL)^4,5^. In eQTL analyses, the main goal is to associate variants located throughout the genome with expression levels of each gene, providing information about the genetic regulation of gene expression in a given tissue. This assists in revealing metabolic pathways, regulatory genes, biological processes, and genetic factors that may be affected by the eQTL, and can modulate a phenotype^6,7^.

Although advances in high-throughput genotyping have made it possible to increase eQTL discovery, the high cost of doing so is still a refraining factor, although genotype imputation can be a powerful tool to reduce costs. This is possible by genotyping a large population of animals with a lower density panel and imputing the genotype of untyped loci using information from a small reference population genotyped with a higher density panel^8,9^. This allows for a larger dataset, increasing the possibility of identifying the regulatory variant associated with gene expression. Another tool that can provide more information by improving the fine-mapping of causal gene regulatory variants is Assay for Transposase-Accessible chromatin (ATAC-seq), as it can identify open chromatin regions^10,11^.

We combined RNA-seq data and imputed genotypes to identify eQTLs. The significant eQTLs were associated with IMF and BFT to identify gene expression regulatory mutations that could modulate phenotypic variance. However, due to linkage disequilibrium, the eQTL may not be the causative variant, so we used ATAC-seq data to select eQTLs associated with the phenotypes that are in open chromatin regions. As a result, we identified six putative variants that modulate gene expression and are associated with IMF and BFT.

## Results

### eQTL analysis with the complete SNP dataset and SNP annotation

Aiming to expand our SNP database to detect regulatory variants, we combined genotypes from 778 progenies (BovineHD BeadChip 770k, Illumina), DNA-Sequencing from 26 Nellore sires of the population under study, and transcribed SNPs called from RNA-Sequence data from the *Longissimus thoracis* (LT) muscle of 192 animals within the progenies. This integration yielded an extensive imputed SNP panel, encompassing a total of 4,522,914 SNPs. Following stringent quality control measures (including minor allele frequency > 5% and call rate > 95%) and pruning (with an r^2^ threshold of 0.8), we refined the SNP dataset for subsequent eQTL analysis, resulting in a set of 553,581 tag-SNPs.

**Figure 1:**
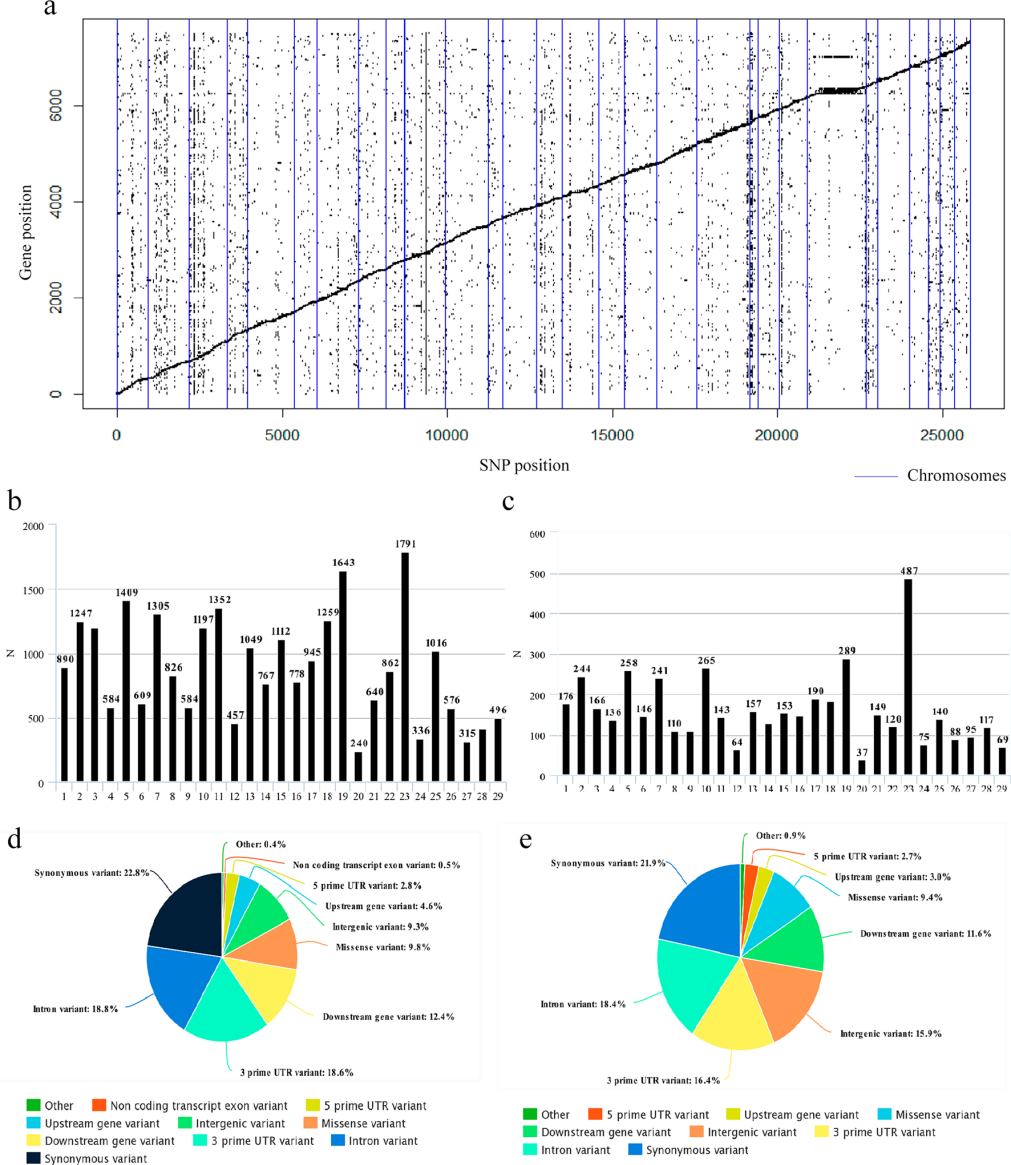
Cis and trans-eQTL distribution along the 29 Bos taurus autosomal chromosomes of a Nellore cattle population. (**a**) Scatter plot of affected genes and eQTL (FDR < 0.05). Y-axis is representing gene order and X-axis is representing SNP order in relation to chromosome position in the genome. Diagonally scattered points indicate cis-eQTL. Vertically scattered points indicate trans-eQTL. Vertical blue lines denote individual autosomal chromosomes. (**b**,**c**) Distribution of cis (**b**) and trans-eQTLs (**c**) across the chromosomes. X-axis is the chromosomes, and Y-axis is the number of variants per chromosome. (**d**,**e**) Most severe consequences of the cis (**d**) and trans-eQTL (**e**) predicted by the Ensembl Variant Effect Predictor.

Before eQTL identification, population stratification was investigated by principal component analysis (PCA). Using 192 animals and the SNPs from the BovineHD BeadChip 770k panel, the first two principal components (explaining 10.64% and 6.87% of the variance for PC1 and PC2, respectively) were included as covariates in the eQTL analysis model, as we observed a sample grouping partially explained by sire (Supplementary Table 1 and Supplementary Fig. 1). Our eQTL analysis resulted in 51,324 eQTLs (FDR < 5%, considering that each time a SNP regulates a distinct gene, it qualifies as an eQTL). There were 36,916 cis-eQTLs (25,896 for a unique genomic position), and 14,408 trans-eQTLs (4685 for a unique genomic position) distributed along the 29 Bos taurus autosomal chromosomes (Fig. 1a). Of these, 3823 SNPs act as cis and trans-eQTLs (1950 for a unique genomic position). From our 25,896 cis-eQTLs and 4685 trans-eQTLs, 2381 and 663 were novel SNPs, absent from the public SNP database (dbSNP), respectively. As for the regulated genes, 5142 genes were affected by cis-eQTLs, and 4707 genes had their expression affected by trans-eQTLs. A quantile–quantile plot illustrating the p-values from the eQTL analysis and a Manhattan plot for both cis and trans-eQTLs is provided in Supplementary Fig. 2. A complete list of cis and trans-eQTLs (FDR < 0.05) and the genes regulated by them can be found in Supplementary Table 2.

Ensembl Variant Effect Predictor (VEP) analysis showed that more cis and trans-eQTL were located on chromosome 23, while fewer local and distant variants were on chromosome 20 (Fig. 1b,c). Cis-eQTLs were predominantly located in 3'UTR, intronic, and downstream gene regions, while trans-eQTLs concentration was in 3'UTR, intronic, and intergenic regions. Among them, 23.8% and 21.9% were predicted to be synonymous variants, whereas 10.2% and 9.4% were classified as missense for cis and trans-eQTL, respectively (Fig. 1d,e and Supplementary Table 3).

### Phenotype association analysis, gene annotation, and SNPs in ATAC-Seq peaks

To discover eQTLs associated with phenotypic variation, we conducted a phenotype association analysis. For this, the population was expanded to 374 animals, and after the population stratification test, PC1 (7.89%) and PC2 (6.96%) were included as covariates to account for the sire effect (Supplementary Table 4 and Supplementary Fig. 3).

**Figure 2:**
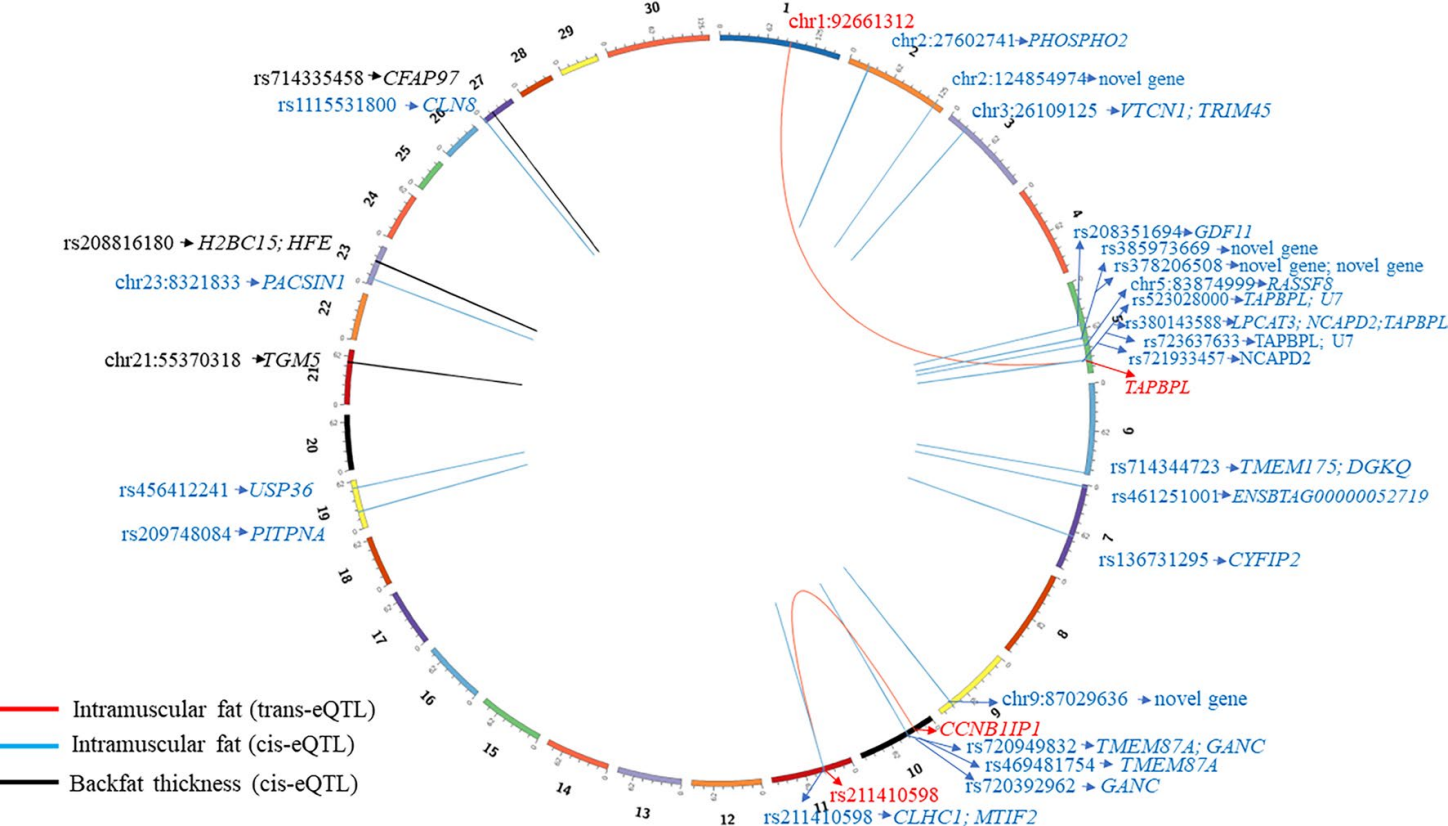
Circos plot of eQTL regions associated with backfat thickness (BFT) and intramuscular fat (IMF) and their regulated genes. Colored boxes represent each chromosome while the colored lines show the association between the regions that harbor the eQTLs and their regulated genes. IMF trans-eQTLs are shown in red, IMF cis-eQTLs are shown in blue and BFT cis-eQTLs are shown in black.

A linear model with the effects of PC1, PC2, contemporary group (CG), and hot carcass weight as covariates was used to test the association of the 30,581 eQTLs (a sum of unique genomic positions of cis and trans-eQTLs) with BFT and IMF. Three eQTLs were associated with BFT (Supplementary Table 5) and 24 with IMF (Supplementary Table 6). The Circos plot shows the links between eQTLs, phenotypes, and their regulated genes (Fig. 2). A quantile–quantile plot and a Manhattan plot illustrating the p-values from the phenotype association analysis is provided in Supplementary Fig. 4.

The biological process of immune response can describe some genes regulated by our eQTLs, including TAP Binding Protein Like (*TAPBPL*), V-Set Domain Containing T Cell Activation Inhibitor 1 (*VTCN1*).). In contrast, Protein Kinase C And Casein Kinase Substrate in Neurons 1 (*PACSIN1*) and Cytoplasmic FMR1 Interacting Protein 2 (*CYFIP2)* are involved in cytoskeleton remodeling. Also, we identified genes related to phospholipid metabolism and lipid biosynthesis, such as Lysophosphatidylcholine Acyltransferase 3 (*LPCAT3*), Phosphatidylinositol Transfer Protein Alpha (*PITPNA*), Diacylglycerol Kinase Theta (*DGKθ*), Growth Differentiation Factor 11 (*GDF11*), and Cytoplasmic FMR1 Interacting Protein 2 (*CYFIP2*). Trans-Glutathione S-Transferase Alpha 2 (*GSTA2*) is involved in oxidative stress, and Homeostatic Iron Regulator (*HFE*) is involved in iron transport pathways. Other genes have previously been associated with meat and carcass quality or are involved in fat-related phenotypes such as Tripartite motif containing 45 (*TRIM45*), Transmembrane Protein 87A (*TMEM87A*), Transmembrane Protein 175 (*TMEM175*), Trans- Aldehyde Dehydrogenase 5 Family Member A1 (*ALDH5A1*), Glucosidase alpha neutral C (*GANC*) and, Non-SMC Condensin I Complex Subunit D2 (*NCAPD2*).

**Figure 3:**
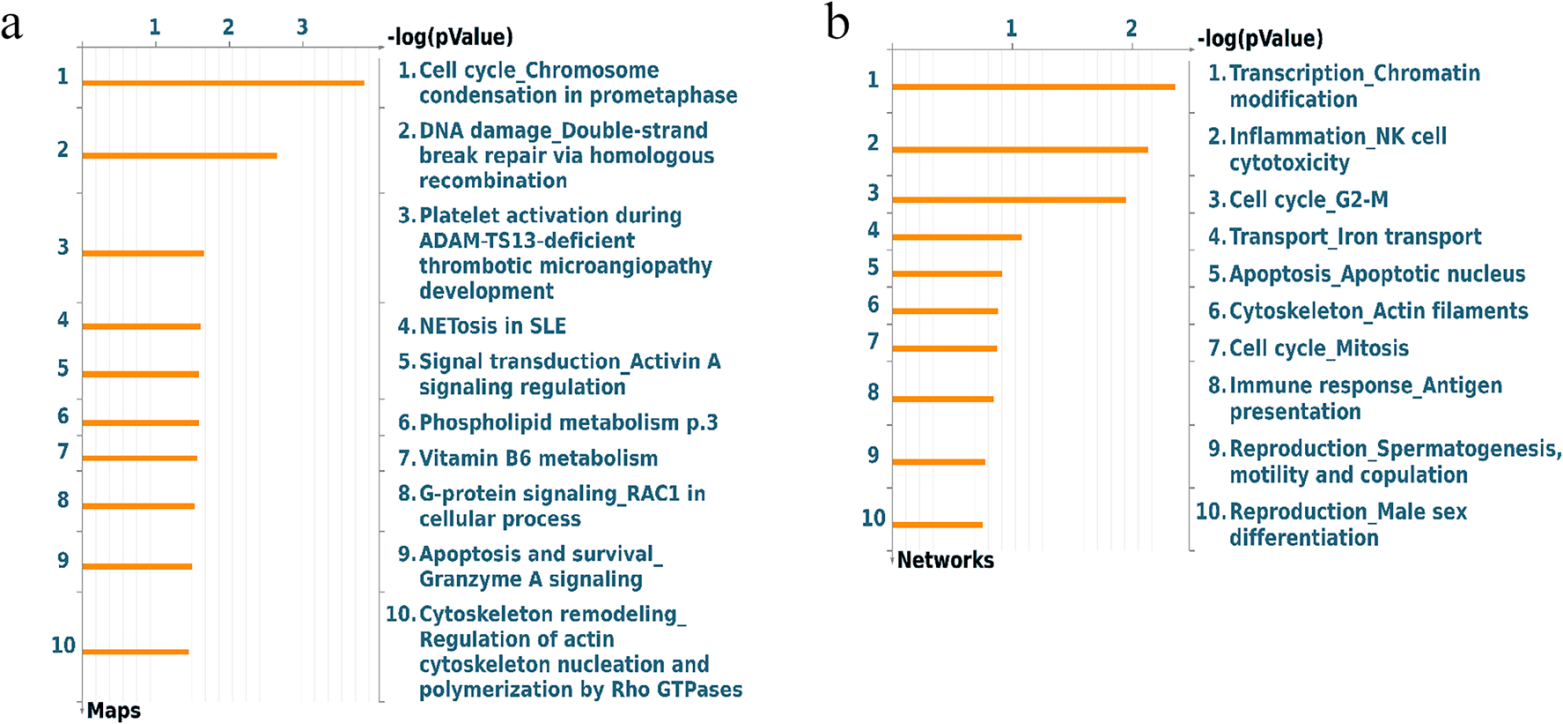
Top ten Pathway Maps (**a**) and the process networks (**b**) enriched for the genes regulated by the eQTLs associated with IMF and BFT in a Nellore cattle population.

Using MetaCore software, we performed an enrichment analysis of the regulated genes. Figure 3 shows the top ten Pathway Maps (Fig. 3a) and the process networks (Fig. 3b) enriched for genes related to IMF and BFT. This revealed some noteworthy pathways, such as immune response, cytoskeleton remodeling, iron transport, and phospholipid metabolism.

To investigate which eQTLs are located at potential regulatory regions, we performed ATAC-Seq in bovine muscle samples. We identified 33,734 ATAC-Seq peaks with an average width of 2193 base pairs (bp) using a Fraction of Reads in Peaks (FRiP) score of 0.2 (Supplementary Table 7). The peaks were selected based on the FRiP score values and were overlapped with Transcriptional Start Sites (TSSs) in each replicate as a quality metric (Supplementary Fig. 5). As expected, the peaks were centralized on TSSs, which indicate high accessibility and, thus, gene expression. We overlapped our 27 traits-associated eQTLs with ATAC-Seq peaks to identify putative causal variants. The overlap resulted in six variants associated with the modulation of gene expression and phenotype, and located in open chromatin regions. Thus, we considered these as candidate regulatory regions (Table 1).

**Table 1:** Relevant eQTLs associated with intramuscular fat (IMF) and backfat thickness (BFT) located in open chromatin regions in a Nellore cattle population.

rsID	CHR	Position	Gene sequence consequences	ATAC-Seq peak window	*Cis* regulated genes	FDR	Phenotype
Novel SNP	21	55370318	5’ UTR variant of *PDIA3-201* gene and intron variant of *CATSPER2-201* gene	chr21: 55368993–55371299	*TGM5*	003952	fBFT
rs208816180	23	31876455	Exon variant of H1-2	chr23: 31875786–31877998	*HFE; H2BC15*	002021	iBFT
rs721933457	5	103915474	5’ UTR variant of *VAMP1* gene	chr5: 103914423–103916378	*NCAPD2*	006543	IMF
rs523028000	5	103856234	Upstream gene variant of *IFFO1*gene	chr5: 103855741–103857330	*TAPBPL; U7*	006543	IMF
rs469481754	10	37638576	Upstream gene variant of *TMEM87A* gene and 5' UTR variant of *GANC* gene	chr10: 37636085–37639779	*TMEM87A*	006543	IMF
rs456412241	19	53553145	3' UTR variant of *USP36* gene	chr10: 37636085–37639779	*USP36*	0.08356	IMF

*rsID* variant identifier, *CHR* SNP located chromosome, *Position* SNP position in Mb, *ATAC-Seq peak window* position in Mb of the peak containing the eQTL, *FDR* phenotype associated False Discovery Rate, *iBFT* initial backfat thickness, *fBFT* final backfat thickness, *IMF* intramuscular fat.

**Figure 4:**
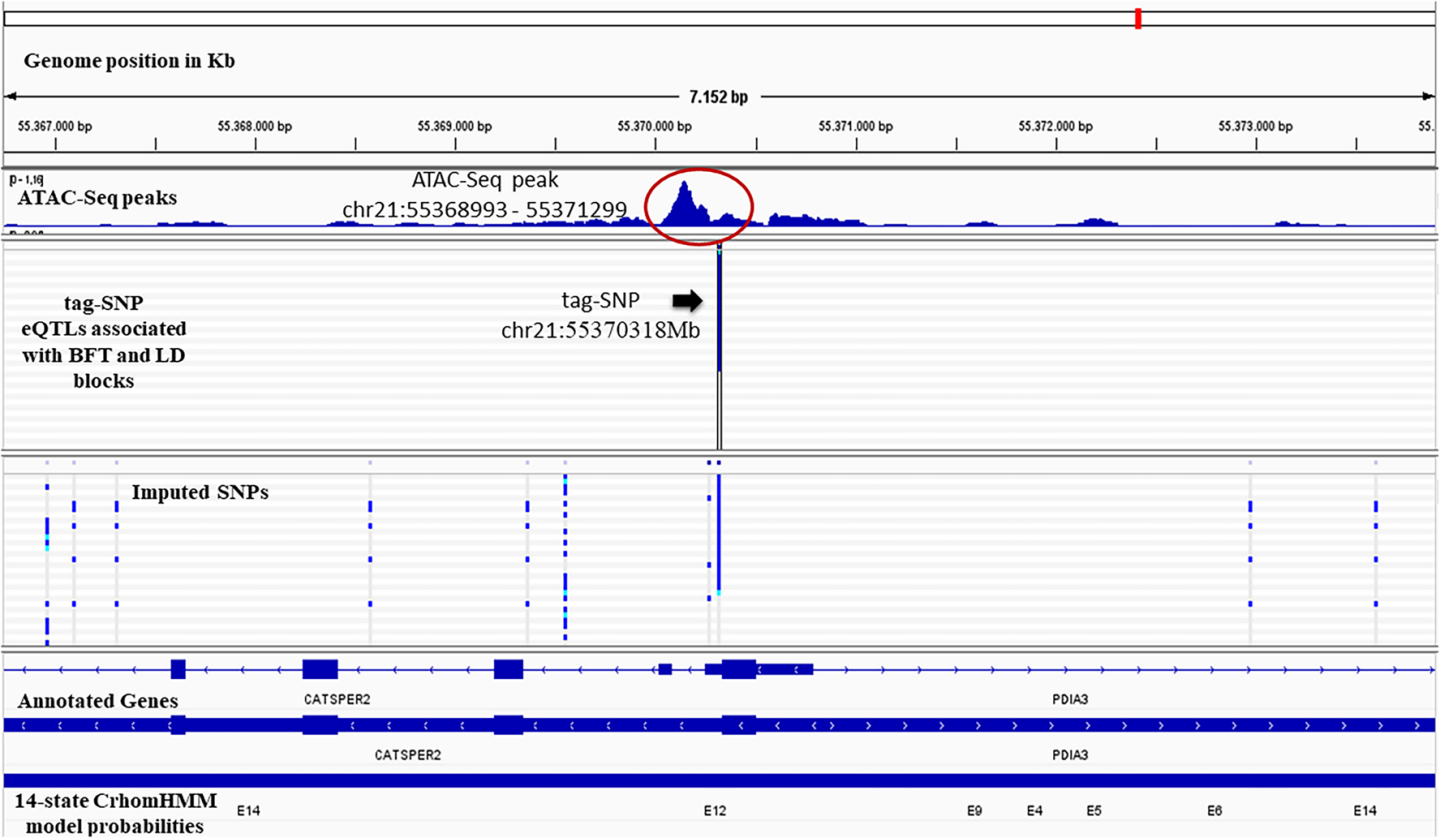
Visualization of the genome location of novel SNP chr21:55370318 on IGV software. The SNP chr21:55370318 is highlighted as "tag-SNP'' with an arrow; the ATAC-Seq Peak is highlighted in a red circle; the 14-state ChromHMM model probabilities for cattle muscle were obtained from^12^, the E12 state predicts an Insulator. The tracks represent the genome position in kb; the ATAC-Seq peaks; the tag-SNPs representing our eQTLs associated with the studied traits; the *Bos taurus* annotated genes (Ensembl Genes); and, the 14-state of chromatin based on Kern et. (2021) according to the figure legends.

In addition to overlapping our variants with ATAC-Seq peaks, we also consulted the ChromHMM model-based profile of chromatin states for cattle muscle (Functional Annotation of Animal and Genomes consortium database—FAANG). This model uses known epigenetic signals of histone marks and CTCF sites to characterize regulatory elements along the chromatin^12^. Figure 4 illustrates the genome location of a novel SNP on chr21:55370318 present in a predicted insulator. Visualization in The Integrative Genomics Viewer (IGV) of the other five eQTLs, located in peaks, can be seen in Supplementary Figs. 6, 7, and 8. Three of those five eQTLs, rs208816180, rs721933457 and rs469481754, are in regions predicted to be insulators. The rs523028000 and rs456412241 eQTLs are in a region with low signal and active enhancer, respectively.

## Discussion

Our research group focuses on unraveling the genetic mechanisms underlying fat deposition in beef cattle. To achieve this, our prior investigations have employed a multi-faceted approach, utilizing Genome-Wide Association Studies (GWAS) to pinpoint relevant genomic regions^13^, RNA-seq analysis to characterize gene expression profiles in animals exhibiting divergent IMF levels^14^, and eQTL analysis to identify variants influencing gene expression within QTLregions^14^.

Herein, we expanded our search for variants by combining genotypes from 374 progenies, whole genome DNA-Sequencing of 26 Nellore sires, and transcribed SNPs obtained from RNA-Seq data from LT of 192 animals to generate a total of 4,522,914 SNPs inputted across 374 animals. The number of SNPs discovered considerably increased our chances of finding causative mutations. However, we had to use linkage disequilibrium to reduce the number of SNPs for eQTL and phenotype association analyses. This reduction in number was essential to decrease the number of multiple tests from SNPs capturing the same effect, thus reducing our analyses to 553,581 tag-SNPs. We also used ATAC-Seq from muscle to localize open chromatin regions. The combination of phenotype association analysis, eQTL, and ATAC-Seq data allowed us to identify mutations in open chromatin regions that control gene expression and are associated with fat deposition.

Most eQTLs identified were in 3'UTR, introns, intergenic, and downstream gene regions. These results corroborate the hypothesis that untranslated gene regions are involved with gene expression regulation^15^. It is essential to point out that the SNPs identified in this study may not be the actual causal mutation since any SNP within the same LD block has the potential to be the causal variant^16^. Our association analysis uncovered 27 eQTLs associated with phenotypic variation, in agreement with previous results on the importance of regulatory mutations for phenotypic variation^14,17^.

When exploring our set of 27 eQTLs in the cattle Genotype-Tissue Expression atlas (cGTEx—https://cgtex.roslin.ed.ac.uk/), we identified four cis-eQTLs associated with intramuscular fat (IMF), confirming our results. These include rs380143588 (chromosome 5), rs378206508 (chromosome 5), rs385973669 (chromosome 5), and rs209748084 (chromosome 19). Notably, none of the eQTLs associated with backfat thickness (BFT) were identified in cGTEx. It is important to note that although certain eQTLs were not detected in this database, a subset of them comprises novel SNPs that have not yet undergone annotation. Additionally, considering that many of our eQTLs associated with IMF and BFT originate from RNA-seq data, SNPs in regulatory regions may not be comprehensively annotated in existing databases, which predominantly consist of SNPs in intronic and more conserved regions, primarily derived from SNP chips.

Three cis-eQTLs were associated with BFT. Among these, a novel SNP (chromosome 21:55370318) was found to modulate the expression of the Transglutaminase 5 (*TGM5*) gene. *TGM5* belongs to the transglutaminase family (TGs), a group of calcium-dependent enzymes responsible for posttranslational modification of proteins through deamidation and amine incorporation processes. These proteins also serve as scaffolds, maintain membrane integrity, regulate cell adhesion, and modulate signal transduction^18,19^. *TGM5* was associated with high triglyceride levels in humans^20^, although this gene's mode of action is unknown. Another member of the TGs family, *TGM2*, a more extensively studied gene, has been indicated as a novel negative regulator of adipogenesis^21^. In addition, higher expression levels of *TGM2* were associated with animals of low genomic estimated breeding values (GEBV) for IMF in a previous study of our group with this same Nellore population^17^.

The cis-eQTL rs208816180, associated with BFT, regulates the expression of *H2BC15* and *HFE*. The first gene, present in a small histone gene cluster, is a histone H2B family member involved in DNA replication^22^. The second gene, *HFE*, regulates iron (Fe^2+^) absorption by mediating the interaction of transferrin receptors with transferrin. This enables the protein to competitively bind to the transferrin receptor at the same site as iron-loaded transferrin molecules, preventing iron uptake^23^. Previous studies suggest that reduced lipogenic activities can occur with lower iron content in the muscle of Nellore steers^24^. This might happen because iron is essential to the adipocyte's physiological functions, such as differentiation, endocrine, and energy supply^25^. Another study discusses how HFE knockout mice can alter lipid metabolism by modifying the expression of lipid metabolism markers^26^.

The last gene regulated by a cis-eQTL associated with BFT is Cilia And Flagella Associated Protein 97 (*CFAP97)*, which is predicted to be involved in spermatogenesis^27^. Although its function in adipose tissue is poorly understood, this gene was associated with adiposity traits such as percent of intramuscular fat, abdominal fat, and blood lipid levels in broilers^28^.

Regarding IMF, 24 eQTLs were associated, regulating 28 genes (six being novel genes). From these, five genes are more directly involved in phospholipid metabolism or lipid biosynthesis, such as *LPCAT3*, *PITPNA*, *DGKθ*, *GDF11* and, *CYFIP2*. *LPCAT3* is an isoform of lysophatidylcholine acyltransferases, which participates in phosphatidylcholine (a major phospholipid class) remodeling^29^. This gene is the main isoform in primary metabolic tissues, such as the skeletal muscle. It has an important role in lipoprotein production and lipogenesis. In contrast, its deficiency reduces lipid absorption^30,31^. *LPCAT3* is also involved in the lipid organization of the plasma membrane and insulin sensitivity in skeletal muscle. Knockout of *LPCAT3* increased plasma membrane lipid clustering and reduced phospholipid packing of cellular membranes, suggesting that this gene modulates plasma membrane lipid organization^32^.

*PITPNA* encodes a family of lipid-binding proteins that transfer molecules of phosphatidylinositol or phosphatidylcholine between membrane surfaces^33^. One isoform of Diacylglycerol Kinase, *DGKθ,* is a transferase that generates phosphatidic acid (another phospholipid involved in cell signaling) by catalyzing the phosphorylation of diacylglycerol using ATP as the phosphate donor, having a role in lipid homeostasis^34^.

Growth Differentiation Factor 11 (*GDF11*) is a member of the Transforming Growth Factor β superfamily. This gene has been reported to be involved in adipogenesis by reducing lipid deposition^35^. Also, *GDF11* inhibits peroxisome proliferator-activated receptor γ (PPAR-γ) expression, one of the central genes involved in adipocyte differentiation^36^.

A recent study in cultured cells reported that *CYFIP2* is involved in thermogenesis and regulation of lipid metabolism in white adipocytes, explicitly influencing the browning of white adipocytes^37^. Other studies reported the possibility of the *CYFIP2* locus being associated with diet-induced obesity and metabolic dysfunction in mice^38,39^. *CYFIP2* was also among the top 10 novel candidate genes for obesity in humans in another study^40^.

The *GSTA2* gene can also play a role in fat deposition and obesity, as this gene is related to oxidative stress, producing an antioxidant enzyme that reduces lipid peroxidation^41^. Oxidative stress can stimulate white adipose tissue deposition and alter food intake, triggering obesity. Studies demonstrated that oxidative stress can increase preadipocyte proliferation, adipocyte differentiation, and mature adipocyte size^42,43^. *GSTA2* may also interact with CCAAT/enhancer binding protein-β (C/EBPβ) which is a fat deposition promoter^44^.

Some identified regulated genes are related to immune response and cytoskeleton remodeling pathways. *TAPBPL* and *VTCN1* are involved in antigen presentation, allowing immune cells to monitor the body for intracellular infection^45,46^. *H2BC15* also has its expression regulated by several immune stimuli in fish, which is probably related to its relevance to immune response^47^. *PACSIN1* can transform the liposome membrane into tubes with different diameters, generating various membrane morphologies^48,49^. *CYFIP2* protein is also a component of the WAVE regulatory complex (WRC) that controls actin cytoskeleton organization^50^.

Other regulated genes have already been associated with meat quality. The *TRIM45* gene regulates cell proliferation, growth, and apoptosis and was highlighted in Gene Ontology (GO) terms, linked to meat quality^51^. In another study, analyzing gene expression by RNA sequencing in LT muscle of Maremmana and Chianina cattle breeds, *TRIM45* was identified as a gene overexpressed in tender Chianina meat^52^. *TMEM87A* and *TMEM175* are part of a huge family of transmembrane protein genes (TMEM). Variants in TMEM genes were associated with obesity (and body fat traits) in human adults and children^53,54^. Moreover, a previous work with this Nellore population found *TMEM39B* as a hub gene in co-expression networks associated with intramuscular fat content traits^4^.

The *ALDH5A1* gene, which is related to glycolipid metabolism pathways, has been identified to play roles in IMF deposition^55^. *GANC* is an enzyme in glycogen metabolism, which is involved in the hydrolysis of this polysaccharide. This gene was linked to energy, carbohydrates, and lipid metabolism pathways. It was also identified as a candidate gene to explain differences in intramuscular fat observed between divergent lines of domestic rabbits^56^. *NCAPD2* enables histone binding activity and is involved in mitotic chromosome condensation and segregation^57,58^.

Our enrichment analysis revealed pathways related to lipid metabolism. Regarding the phospholipid pathways, lipid intermediates, such as diacylglycerol and fatty acids released from phospholipids, play a crucial role in triglyceride synthesis within the body^59,60^. Additionally, we identified genes associated with immune response and cytoskeleton remodeling. One of the consequences of obesity is the presence of chronic inflammation in adipose tissue. When there is an increase in adipocyte volume, the enlarged adipocyte begins producing proinflammatory chemokines, which in turn recruit immune cells to the tissue^61,62^. Also, when the adipocyte enlarges to store lipids, the cell must undergo a remodeling of its cytoskeleton^63^. This means that fat deposition, immune response, and cytoskeleton remodeling are associated, and with a gain of adipose tissue mass, genes and pathways related to immune response and cytoskeleton remodeling are likely upregulated.

Some of the genes regulated by the six eQTLs located in ATAC-Seq peaks in our study have been associated in the past with fat-related phenotypes, having their expression regulated by other variants^14,20^. An example of this is the eQTLs rs721933457 and rs523028000, which in our study, regulate *NCAPD2* and *TAPBPL* respectively. In another study with our current Nellore cattle population, these genes were regulated by different eQTLs that were associated with IMF^14^. Also, another variant located in *TGM5* was associated with high triglyceride levels^20^, but in our study the eQTL that regulates this gene is a novel SNP (chromosome 21:55370318). In these cases, ATAC-Seq data can help us determine that the eQTLs rs721933457, rs523028000 and novel SNP (chromosome 21:55370318) are more likely the putative causal mutations, as this analysis prioritizes variants located in regulatory regions.

When considering the regions where the eQTLs were found within ATAC-Seq peaks, integrating multiple epigenomic marks provides further insights into their biological relevance within a spatial context; these regions are commonly referred to as chromatin states^64^. Chromatin states can be used to identify different genomic elements, such as active enhancers, transcription start sites, and insulators. This allowed us to identify regulatory regions and better explain gene expression regulation^65^. When visualizing the eQTLs located in ATAC-Seq peaks in IGV, four of them were co-located within regions where the chromatin state was predicted to act as insulators, alongside potential CCCTC-binding factor (CTCF) binding sites. Insulators are regions in the DNA where some elements can bind to protect the expression of genes from signals emitting from their surrounding regions. Insulators can protect gene expression in two ways: by acting as an enhancer-blocking element or by functioning as a barrier, preventing the advance of condensed chromatin that could silence expression^66^. CTCF are highly conserved zinc finger proteins that act as a transcription factor by activating and repressing gene expression or by acting as an insulator protein. CTCF can recruit other transcription factors while binding to chromatin domain boundaries, preventing the advance of condensed chromatin and creating an open chromatin region^67,68^. An eQTL located in a cis-regulatory element such as insulators can, in turn, affect gene expression^69^.

SNPs rs523028000 and rs456412241 are in a region with low signal and active enhancer, respectively. An active enhancer is a cis-regulatory region where some elements, such as transcription factors, can bind to regulate the intensity of gene transcription^70^.

In conclusion, by integrating different methods, we could identify putative regulatory variants that are associated with BFT and IMF. Most of our genes and pathways regulated by eQTLs are directly or indirectly involved in lipid metabolism and fat deposition. Furthermore, we identified eQTLs that are in open chromatin regions, colocalized with a profile of noteworthy chromatin states, which assist us in pinpointing candidate regulatory variants. This finding can contribute to improving livestock traits of economic relevance by helping us unravel how these regulatory genomic variants affect fat traits in Nellore cattle.

## Materials and methods

### Animals, samples, and phenotypes

Experimental procedures related to animal handling and care were approved by the Institutional Animal Care and Use Committee Guidelines of the Brazilian Agricultural Research Corporation (EMBRAPA) (CEUA 01/2013). All methods were performed in accordance with the relevant guidelines and regulations. This study was carried out in compliance with the ARRIVE guidelines.

We used a population of 374 Nellore steers derived from an experimental herd of EMBRAPA, that originated from 34 unrelated bulls representing the main Brazilian Nellore genealogies. Between the years 2009–2011, animals were raised in grazing systems and finished in feedlots with the same handling and nutritional conditions. Steers were slaughtered at an average age of 25 months and 452 kg in a commercial slaughterhouse located in Bariri (São Paulo, Brazil), following the Brazilian Ministry of Agriculture, Livestock and Food Supply (MAPA) guidelines. For additional details see^71,72^.

A muscle sample from the *Longissimus thoracis* (LT) was collected for RNA extraction. Approximately 5 g was collected from the right side of each carcass between the 12th and 13th ribs immediately after the animal's death and stored at − 80 °C until analysis. At the beginning of the feedlot period, initial backfat thickness (iBFT, mm) was measured on the animal's back, between 12th and 13th ribs using a Pie Medical Aquila ultrasound device (Pie Medical, Inc. Maastricht, The Netherlands) equipped with a 17-cm 3.5 MHz transducer^73^. For measurements of intramuscular fat content (IMF, %) and final backfat thickness (fBFT, mm), a beef sample of the LT muscle (12th–13th ribs, left side of the carcass) was collected 24 h after slaughter. For IMF analysis, beef samples of approximately 100 g were lyophilized and ground, then IMF was measured using the Ankom XT20 extractor, following the AOCS protocol^96^, more details have already been described in another study of our group^72^. The fBFT was measured using a graduated ruler, as previously described^71^.

### DNA extraction and high-density genotyping data

Genotyping analysis was performed for the 374 animals at the Bovine Functional Genomics Laboratory ARS/USA and ESALQ Genomics Center (Piracicaba, São Paulo, Brazil). Steers and their sires were genotyped using the BovineHD 770 k BeadChip (Infinium BeadChip, Illumina, San Diego, CA, USA), which included 783,450 SNPs, following Illumina's protocol. The DNA of the steers was isolated from the blood through a salting-out method. As a quality control step, SNPs with call rate ≤ 95%, minor allele frequency (MAF) ≤ 5%, located in sexual chromosomes, and those not mapped in the *Bos taurus* ARS-UCD1.2 reference genome were excluded from further analysis. This analysis was already described in more detail by^72,74^. Sires' DNA was extracted from straws of frozen semen by using a standard phenol–chloroform method; more details can be found in^75^. Whole-genome sequence data of the 26 sires were obtained with the Illumina HiSeq 2500 System (Illumina Inc., San Diego, CA, USA), as previously described by^76^. Briefly, reads were trimmed and filtered using the Trimmomatic v.0.36^77^ program and mapped to the ARS-UCD1.2 Bovine reference genome using the Burrows–Wheeler Aligner (BWA) v.0.7.17^78^. Samtools v.1.8^79^ was used to sort the mapped reads by sequence coordinates. The SNPs were then called using the GATK ‘HaplotypeCaller’.

### RNA-sequencing

RNA-Sequencing data acquisition was already described elsewhere^17^. Briefly, a subset of 192 animals from the 374 animals used for the phenotype association analysis were selected. A sample of 100 mg of the LT muscle was processed using the Trizol reagent (Life Technologies, Carlsbad, CA, USA), following the manufacturer's guidelines. After extraction, RNA integrity was verified using the Bioanalyzer 2100 (Agilent, Santa Clara, CA, USA), and the samples presenting RNA integrity numbers (RIN) greater than 7 were considered for the next analyses. A total of 2 µg of RNA from each sample was used for the cDNA library preparation, according to the protocol described in the TruSeq RNA Sample Preparation kit v2 guide (Illumina, San Diego, CA, USA). The libraries were sequenced using the Illumina HiSeq2500 ultra-high-throughput sequencing system with the TruSeq SBS kit v3-HS (200 cycles), as described in^17^. All sequencing analyses were performed at ESALQ Genomics Center (Piracicaba, São Paulo, Brazil). After sequencing, the SeqyClean package v. 1.4.13 (https://github.com/ibest/seqyclean) was utilized to remove low complexity reads and adapter sequences from the library preparation step. For quality control visualization, FastQC software v. 0.10.1 (https://www.bioinformatics.babraham.ac.uk/projects/fastqc/) was used. The RNA-sequencing dataset analyzed in this study can be found in the European Nucleotide Archive (ENA) repository (EMBL-EBI) under the following accession codes: PRJEB13188, PRJEB10898, and PRJEB19421.

### Variant calling analysis and SNP annotation

SNPs were identified in the gene expression data using the Genome Analysis Toolkit (GATK) program v. 4.1.0.0 in Genomic Variant Call Format (GVCF) mode, following the program's best practices manual^80,81^. Data from 192 animals with RNA-seq information were used to call the variants individually by haplotype, and the Ensembl *Bos taurus* database SNP (release 96) was used as a database of known variants. Identified SNPs that had variant quality based on Phred (Phred scaled polymorphism probability) > 30 and minimum variant coverage > 10, were also filtered for call rate > 95% and MAF (minor allele frequency) > 5%. Non-biallelic SNPs located on sex chromosomes were not considered in the analysis. This variant acquisition step from RNA-Seq data was previously described in another study of our group^14^.

### SNP imputation data

Identified SNPs in the genomic DNA sequence of 26 sires were jointly imputed imputed to 778 progenies with genotypes obtained from the BovineHD BeadChip 770 k panel (Illumina, San Diego, CA, USA), using the programs Eagle^82^ (for phasing) and Minimac3^83^ (for imputation). Imputation accuracy was obtained using leave-one-out cross-validation, in which each sequenced animal was removed once from the reference population and included in the target population along with the progenies genotyped with the high-density panel. Thus, imputation accuracy metrics were calculated by comparing imputed alleles to alleles observed in the DNA sequence of each sire. The allelic imputation error rate was estimated as the ratio between the number of incorrectly imputed alleles and the total of alleles imputed. We also estimated the correlation between imputed and actual genotype. Only SNPs with a correlation greater than 0.98 and allelic imputation error rate < 2% were kept for further analysis. Therefore, positions with low imputation accuracy were discarded. Additionally, only SNPs in autossomol chormossome and with an allele frequency greater than 5% were maintained for the analysis.

The 123,300 SNPs identified in RNA sequencing of 192 animals within the progenies (call rate < 95%) were subsequently jointly imputed to the panel formed by imputed DNA-Seq variants plus SNPs from the high-density panel, using Eagle and Minimac3 programs for phasing and imputation, respectively. After imputation, 96.195 SNPs were kept with R2 (calculated by Minimac3) > 0.90. The SNPs were also filtered for allele frequency, removing monomorphic and SNPs with MAF < 5%. A graphical summary of the imputation analysis can be seen in Supplementary Fig. 9.

### Population stratification test

Population stratification was investigated by principal component analysis (PCA) using the genotypes from the BovineHD 770 k BeadChip and our population of 192 (for eQTL mapping) and 374 animals (for association analysis). We first filtered the variants for MAF > 5% and call rate > 95%. Then, using the PLINK software^84^ we tested the population stratification with 446,498 genotypes and used the sires to test for sample clustering since our population was sired by 34 unrelated bulls.

### eQTL identification and functional annotation

The imputed SNPs were filtered for the subset of 192 animals with RNA-Seq information. Additionally, we performed quality control of the variants keeping only SNPs with MAF > 5% and call rate > 95%, totaling 4,436,504 SNPs. PLINK v. 1.07^84^ was used in the set of imputed SNPs to select these, based on linkage disequilibrium (LD) calculation and pruning of the variants. Parameters applied to variant pruning were pairwise connections with a minimum r^2^ of 0.8 and a window size of 100 SNPs, shifting 10 SNPs in each step, to obtain a subset of informative SNPs (tag-SNPs) within linkage disequilibrium (LD) blocks. The R package, Matrix eQTL v. 2.3^85^, was used to perform cis and trans-eQTL identification, with the expression of 12,991 muscle genes data normalized in log2-CPM (counts per million of mapped reads) and adjusted for lane and flow cell effects; the genotype file containing the tag-SNPs; the first two main principal components (PC1 and PC2), to correct putative effects from population stratification, and CG (animals from the same farm, year, and slaughter date) as covariates in the model. In this study, cis-eQTLs were defined as SNPs located up to 1 Mb away from the regulated gene, while trans-eQTLs were SNPs located > 1 Mb away from the gene. The additive effect of each gene-SNP pair was tested by linear regression and false discovery rate (FDR), based on the Benjamini–Hochberg methodology^86^, was calculated separately for cis and trans-eQTLs^85^. The lists of cis and trans-eQTL (FDR < 5%) were annotated separately using VEP v. 101.0^87^.

### Phenotype association analysis

Association between 30,581 significative eQTLs (FDR < 5%) and the phenotypes were performed in PLINK v. 1.07 software^84^, using a linear model and a SNP-by-SNP approach, with adjustment for multiple tests, and a population of 374 animals, the same used by^13^. This analysis was performed considering the same effects used in the eQTL mapping, being PC1, PC2, and CG. Furthermore, following the model used in a previous study by our research group^13^, hot carcass weight was included in the model as a covariate. SNPs associated with the phenotype at FDR < 10%^86^ were considered significant.

### Gene's annotation and functional enrichment

Annotation of eQTL-regulated genes was performed using the Ensembl Biomart tool (Ensembl Genes 104). To find molecular pathways in which genes regulated by the representative eQTL were involved, we used MetaCore software (https://portal.genego.com/) from Clarivate (London, GBR) with the *Homo sapiens* database and the list of annotated genes.

### ATAC-Seq

We performed ATAC-Seq analysis in two LT samples from Nellore males, purchased from a commercial slaughterhouse, and then replicated in two technical replicates for each sample using the protocol described by^88^. A paired-end sequencing (2 × 100) using HiSeq 2500 was performed, and approximately 40 million reads were generated per library. Trimmomatic (v:0.36)^77^ was used to remove adapters from read ends, using the FastQC tool for quality control^89,90^. Then, we generated ATAC-Seq pileup files from FASTA files using the nfcore/atacseq pipeline implemented in Nextflow (https://nf-co.re/atacseq). A consensus peak mapping was obtained from the four samples by considering only regions with counts higher than zero in all. In these regions, the FRiP score was calculated for each of the replicates, and an ATAC-Seq Peak region was considered when the average FRiP score in the four replicates was higher than 0.2, following the ENCODE consortium (Encyclopedia of DNA Elements) recommendations^91^. The deepTools v.3.5.1^92^ was used to create the matrix, heatmaps and line plots of overlapping peaks with TSS from individual and merged replicates as another quality control^93^.

To explore the genomic overlap between eQTLs associated with the phenotypes and ATAC-Seq peaks, we used an in-house R script based on the *subsetByOverlaps* function from the GenomicRanges R/Bioconductor package^94^. The Integrative Genomics Viewer (IGV – v.2.15.4) was used for data visualization^95^. The ATAC-Seq dataset analyzed in this study can also be found in the ENA repository (EMBL-EBI) under the accession code PRJEB64479.

### Supplementary Information

Supplementary Table 1.

Supplementary Table 2.

Supplementary Table 3.

Supplementary Table 4.

Supplementary Table 5.

Supplementary Table 6.

Supplementary Table 7.

Supplementary Figures.

## Data Availability

The datasets used in this study can be found in online repositories. The RNA-sequencing dataset analyzed in this study can be found in the European Nucleotide Archive (ENA) repository (EMBL-EBI) under the following accession codes: PRJEB13188, PRJEB10898, and PRJEB19421. The Atac-Seq dataset analyzed in this study can also be found in the ENA repository (EMBL-EBI) under the accession code PRJEB64479. Accession: https://www.ebi.ac.uk/ena/browser/view/PRJEB13188; https://www.ebi.ac.uk/ena/browser/view/PRJEB10898; https://www.ebi.ac.uk/ena/browser/view/PRJEB19421; https://www.ebi.ac.uk/ena/browser/view/PRJEB64479.
