# Supplementary material for: Novel putative causal mutations associated with fat traits in Nellore cattle uncovered by eQTLs located in open chromatin regions
Source: Sci Rep. 2024 May 2;14:10094. doi: 10.1038/s41598-024-60703-5 (PMC11066111; doi:10.1038/s41598-024-60703-5)
Supplement: Supplementary file 8 — Supplementary Figures. [file 41598_2024_60703_MOESM8_ESM.pdf]

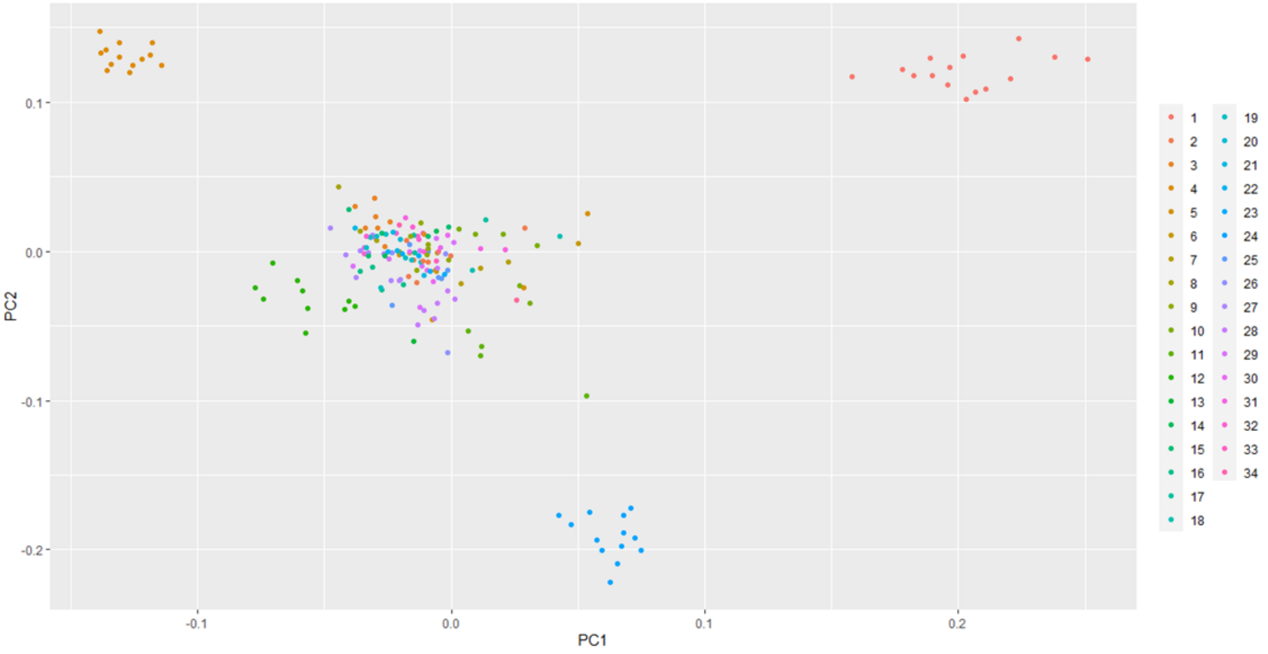

Supplementary Figure 1. Supplementary Figure 1. Principal Component Analysis (PCA) plot representing the genotypes from the BovineHD 770k Bead Chip and our population of 192 Nellore cattle. The plot is colored considering the 34 sires of this population (represented as different numbers in the legend). The X-axis correspond to the first principal component, Y-axis correspond to the second principal component.

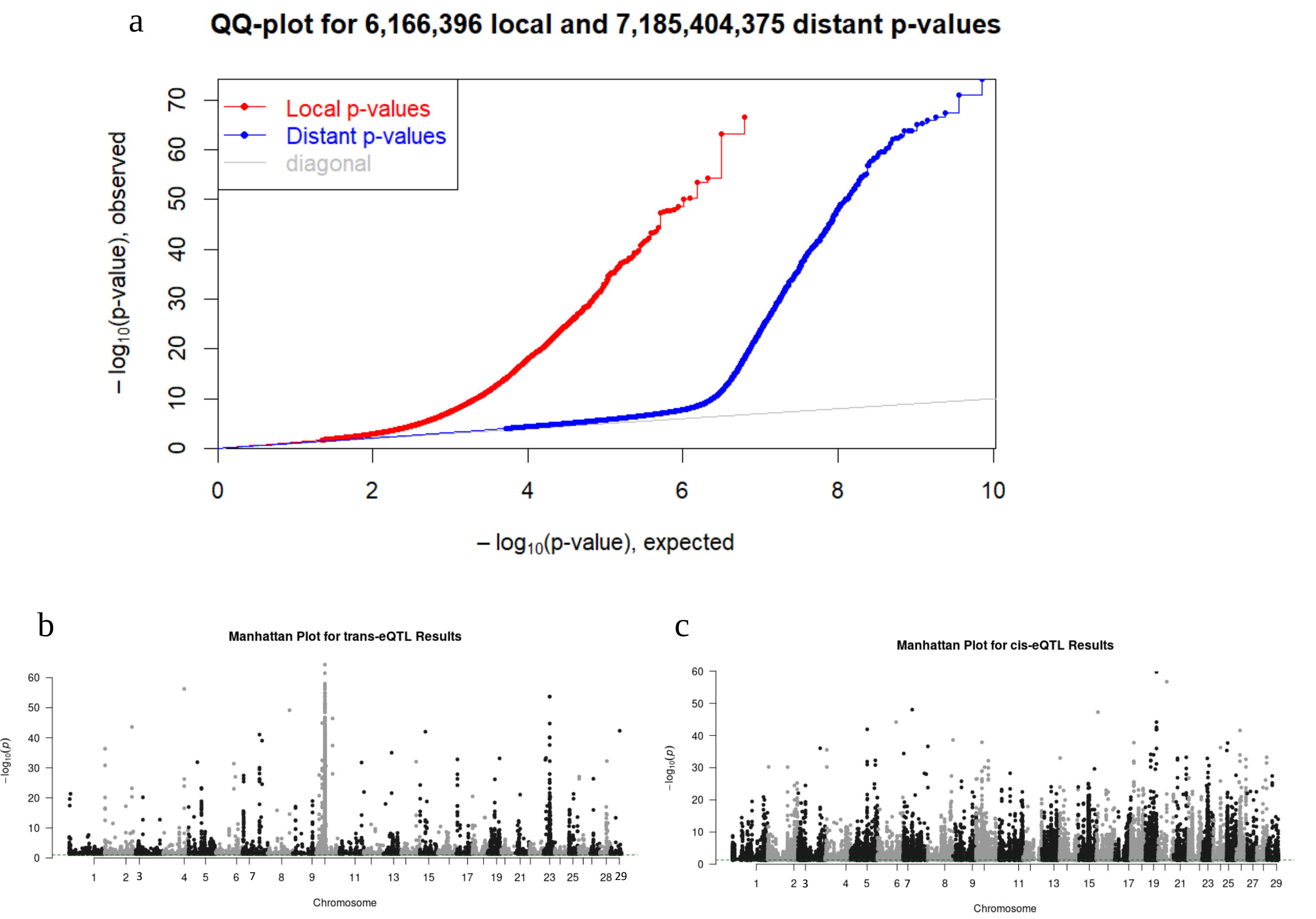

Supplementary Figure 2. (A) Quantile-quantile plot of all local (cis-eQTLs) and distant (trans-eQTLs) p-values illustrating the distribution of expected p-values (X-axis) under a null model of no significance versus the observed p-values (Y-axis). The x-axis represents the expected -log10 transformed p-values for each association, while the y-axis displays the observed values. the expected distribution under the null hypothesis is represented by the diagonal grey line. (B-C) Manhattan plot containing the significant eQTLs (FDR <0.05), showing significance of each variant's association with a gene. Each dot represents a single-nucleotide polymorphism (SNP), with SNPs ordered on the *x* axis according to their genomic position. *y* axis represents their association measured as $-\log_{10}$ transformed *P* values. Green line marks significance threshold of FDR <0.05. (C) Manhattan Plot for trans-eQTLs. (D) Manhattan Plot for cis-eQTLs.

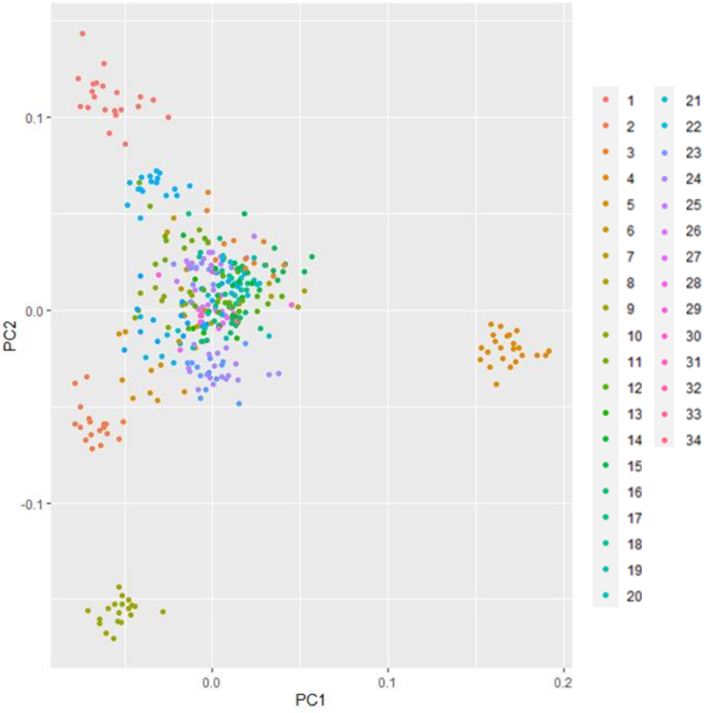

Supplementary Figure 3. Supplementary Figure 2. Principal Component Analysis (PCA) plot representing the genotypes from the BovineHD 770k Bead Chip and our population of 374 Nellore cattle. The plot is colored considering the 34 sires of this population (represented as different numbers in the legend). The X-axis correspond to the first principal component, Y-axis correspond to the second principal component.

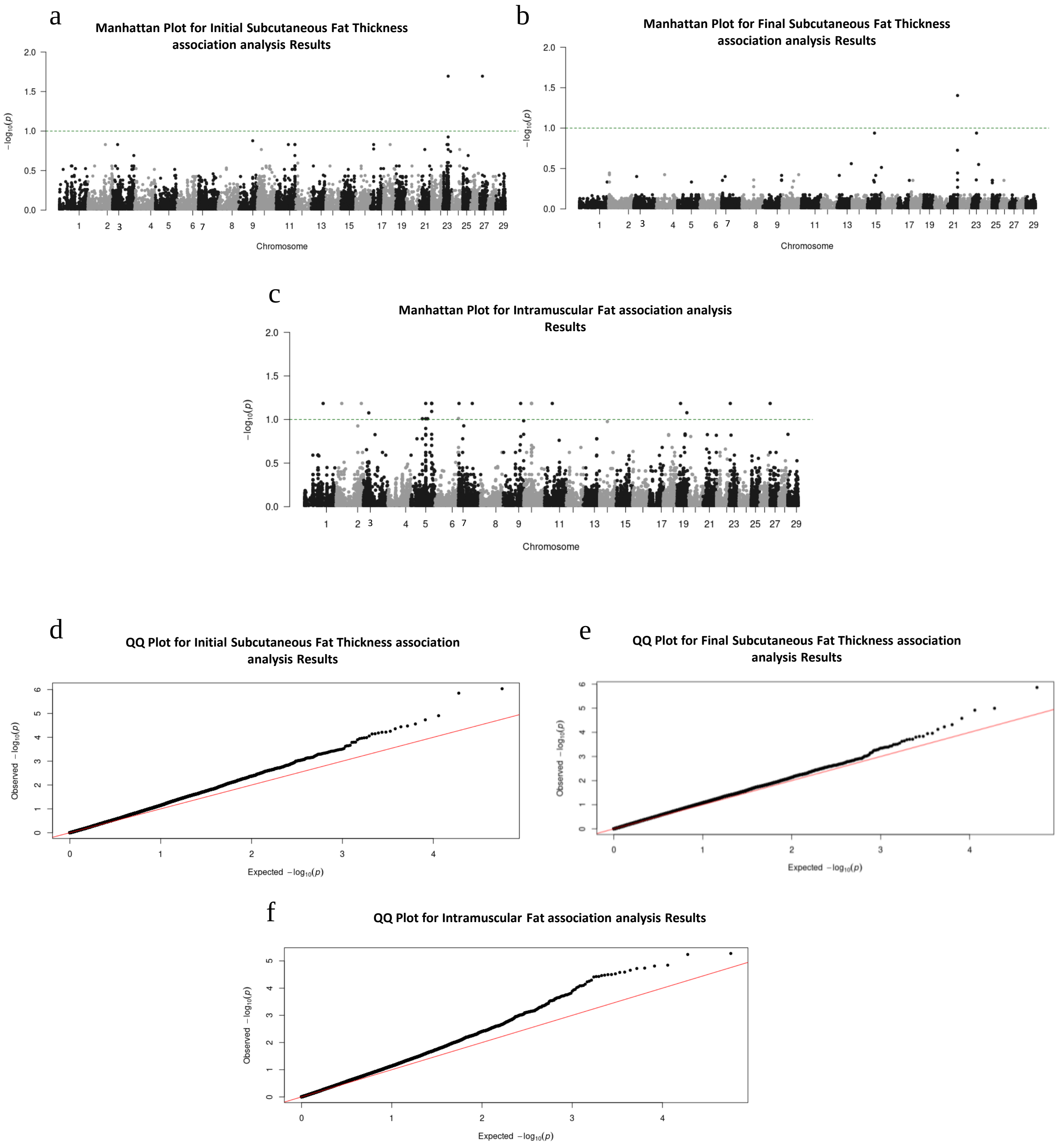

Supplementary Figure 4. (A-B-C) Manhattan plot showing significance of each variant's association with a phenotype. Each dot represents a single-nucleotide polymorphism (SNP), with SNPs ordered on the *x* axis according to their genomic position. *y* axis represents their association measured as –$\log_{10}$ transformed *P* values. Green line marks genome-wide significance threshold of FDR <0.1. (D-E-F) Quantile-quantile plot illustrating the distribution of expected P values (X-axis) under a null model of no significance versus the observed P values (Y-axis). The x-axis represents the expected -log10 transformed P values for each association, while the y-axis displays the observed values. The expected distribution under the null hypothesis is represented by the diagonal red line.

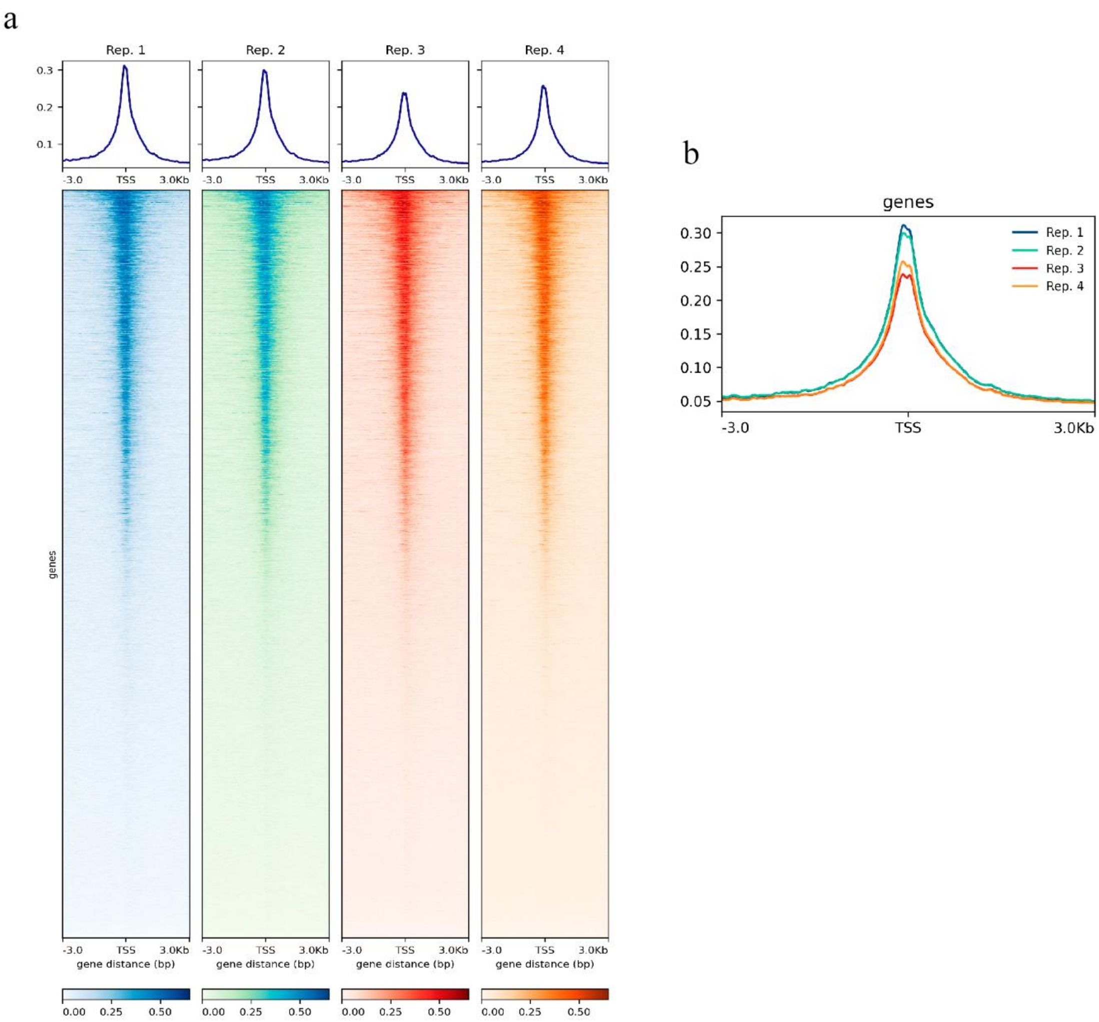

Supplementary Figure 5. Heatmap and composite profiles of ATAC-Seq data around transcriptions start sites (TSS) and annotated genes in the *Bos taurus* genome. (A) ATAC-Seq heatmap signals overlapping TSS of four bovine muscle replicates, dark colors represent ATAC-Seq peaks density (FRIP Score > 0.2). (B) Overlap of all replicates on TSS region. The X-axis represents gene distance in bp, Y-axis represents the FRIP score (A and B).

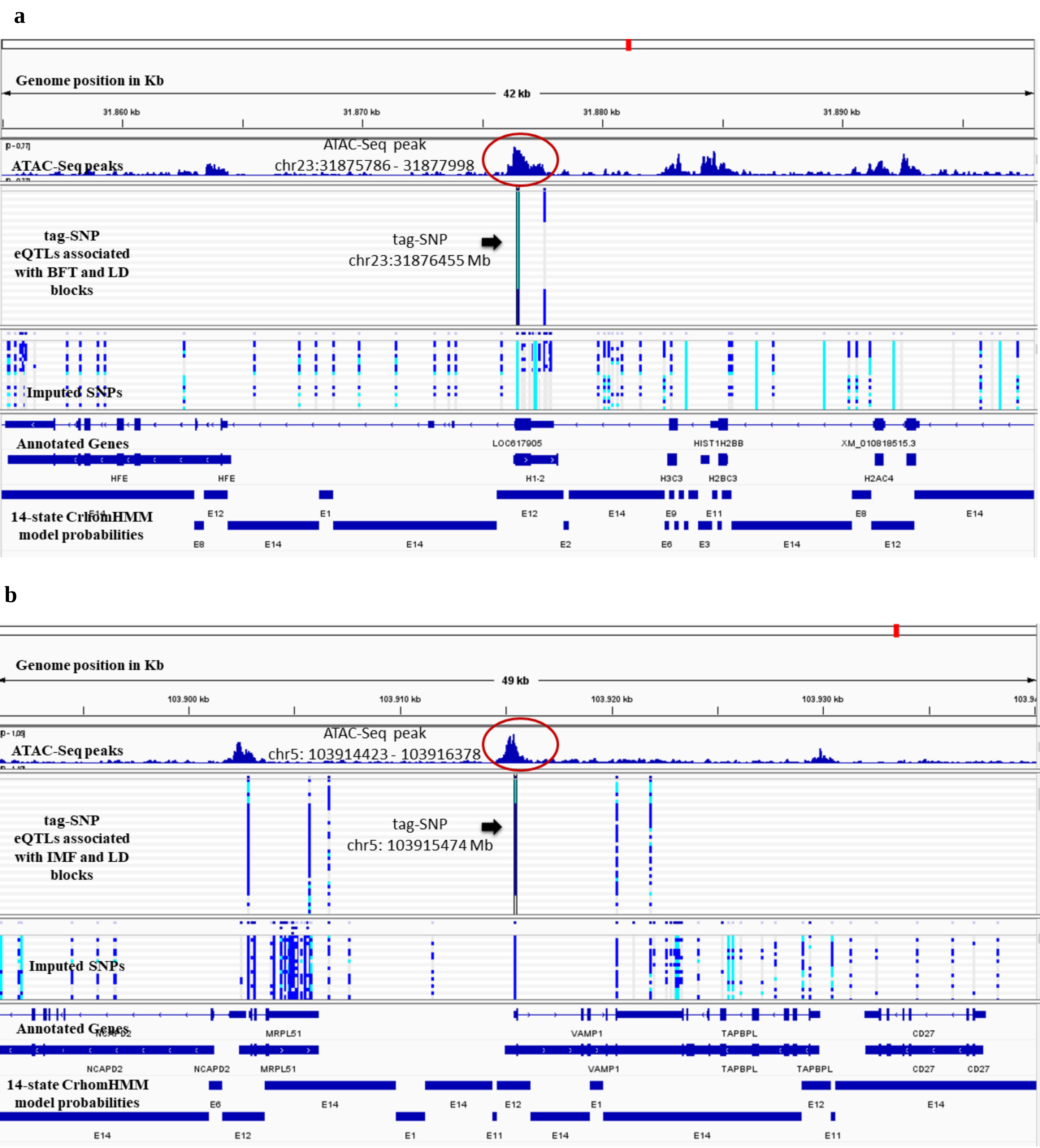

Supplementary Figure 6. Visualization of the genome location of eQTLs generated by IGV software. (A) The SNP rs208816180 is highlighted as “tag-SNP” with an arrow; the ATAC-Seq Peak is highlighted in a red circle; the 14-state ChromHMM model probabilities for cattle muscle were attained from Kern et al. (2021), the E12 state predicts an Insulator. (B) The SNP rs721933457 is highlighted as “tag-SNP” with an arrow; the ATAC-Seq Peak is highlighted in a red circle; the 14-state ChromHMM model probabilities for cattle muscle were attained from Kern et al. (2021), the E12 state predicts an Insulator. The tracks represent the genome position in kb; the ATAC-Seq peaks; the tag-SNPs representing our eQTLs associated with the studied traits; the *Bos taurus* annotated genes (Ensembl Genes); and, the 14-state of chromatin based on Kern et. (2021) according to the figure legends.

A

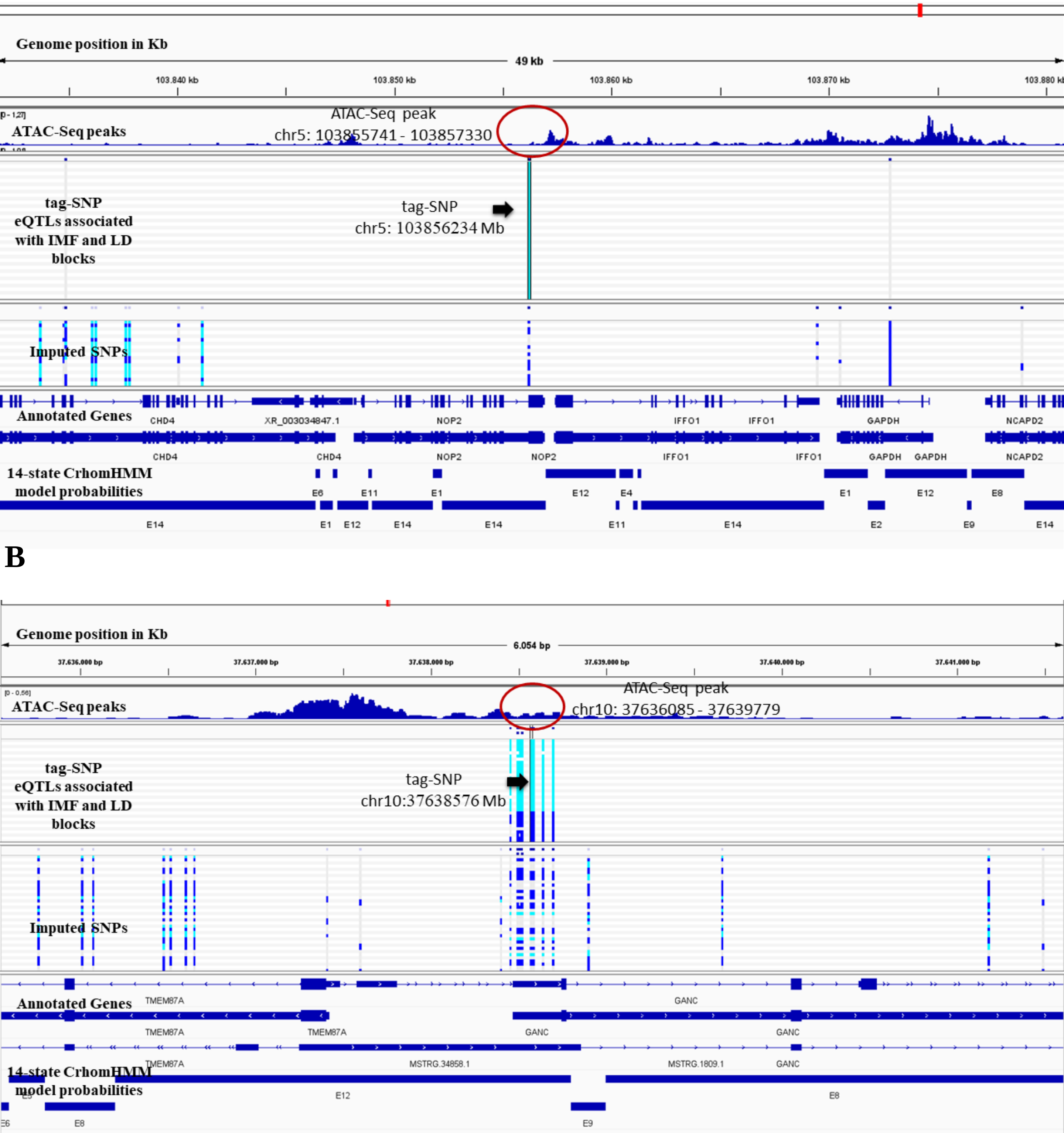

B

Supplementary Figure 7. Visualization of the genome location of eQTLs generated by IGV software. (A) The SNP rs523028000 is highlighted as "tag-SNP" with an arrow; the ATAC-Seq Peak is highlighted in a red circle; the 14-state ChromHMM model probabilities for cattle muscle were attained from Kern et al. (2021), the E14 state predicts a low signal. (B) The SNP rs469481754 is highlighted as "tag-SNP" with an arrow; the ATAC-Seq Peak is highlighted in a red circle; the 14-state ChromHMM model probabilities for cattle muscle were attained from Kern et al. (2021), the E12 state predicts an Insulator. The tracks represent the genome position in kb; the ATAC-Seq peaks; the tag-SNPs representing our eQTLs associated with the studied traits; the *Bos taurus* annotated genes (Ensembl Genes); and, the 14-state of chromatin based on Kern et. (2021) according to the figure legends.

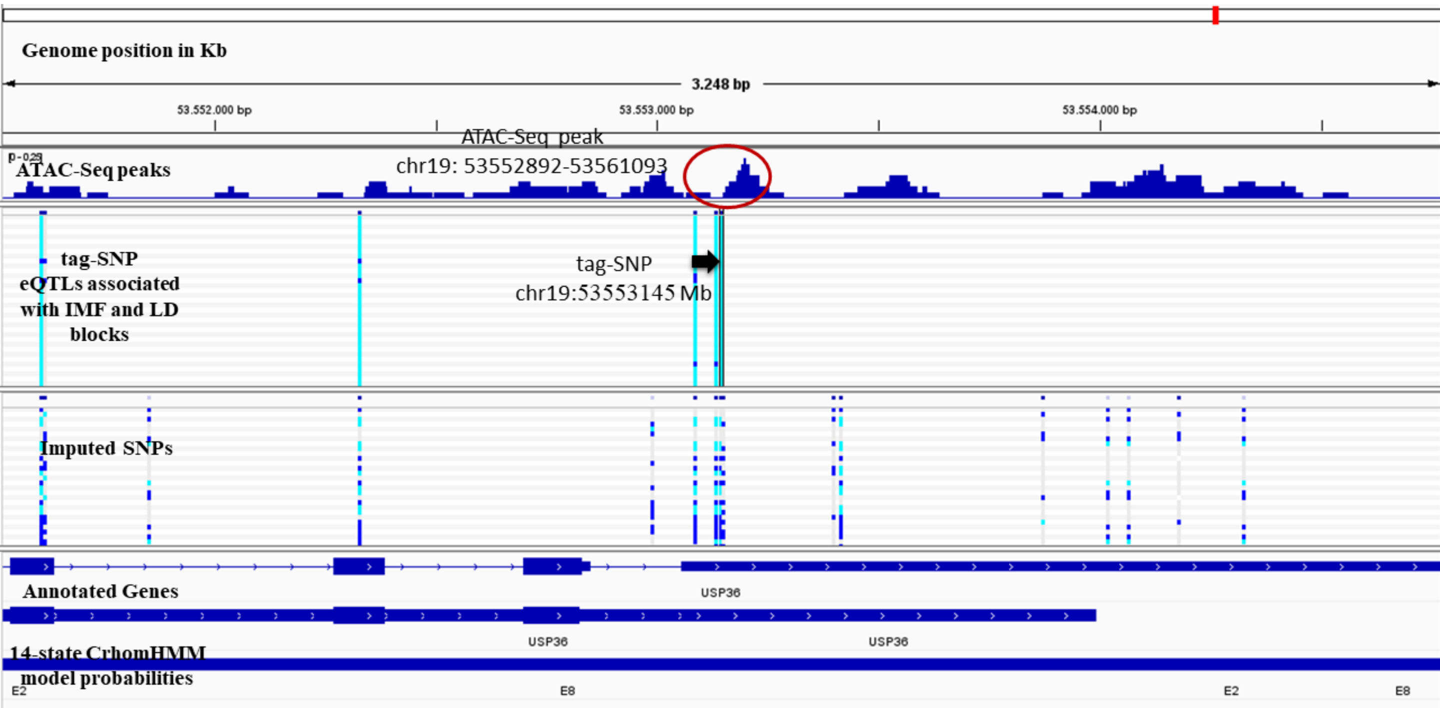

Supplementary Figure 8. Visualization of the genome location of eQTLs generated by IGV software. The SNP rs456412241 is highlighted as "tag-SNP" with an arrow; the ATAC-Seq Peak is highlighted in a red circle; the 14-state ChromHMM model probabilities for cattle muscle were attained from Kern et al. (2021), the E8 state predicts an active enhancer. The tracks represent the genome position in kb; the ATAC-Seq peaks; the tag-SNPs representing our eQTLs associated with the studied traits; the *Bos taurus* annotated genes (Ensembl Genes); and, the 14-state of chromatin based on Kern et. (2021) according to the figure legends.

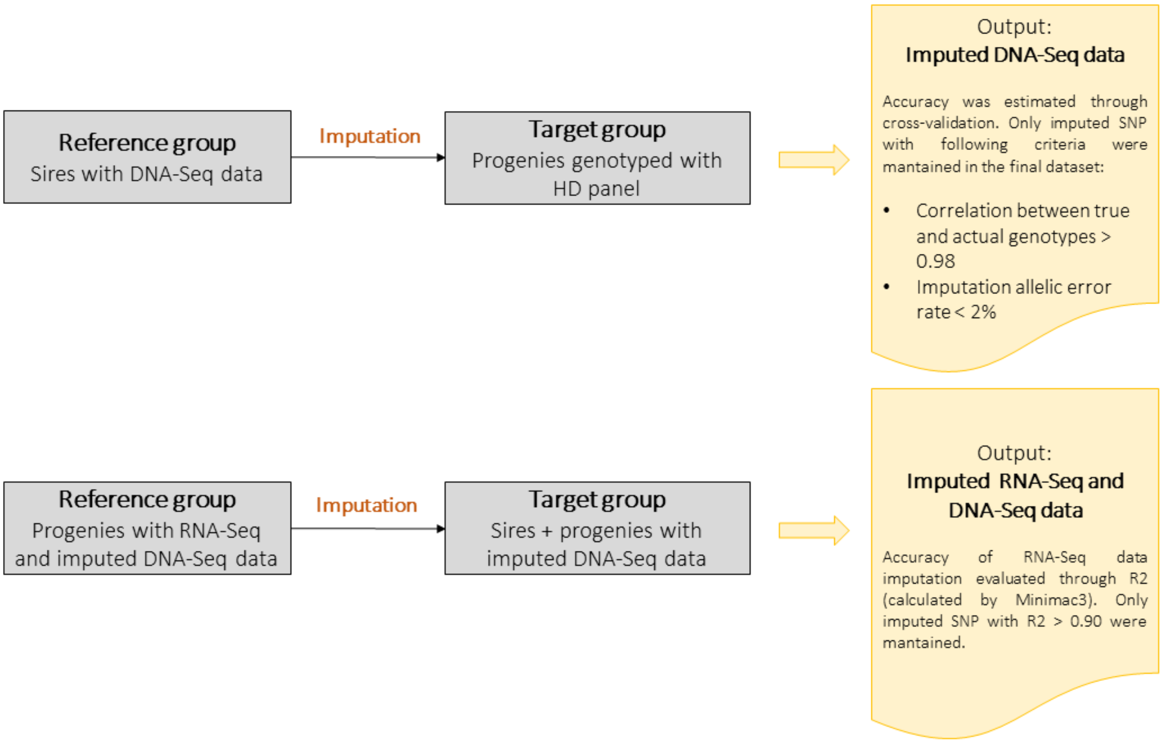

Supplementary Figure 9. Graphical summary of the imputation analysis.
